# The thoracolumbar interfascial block with local anesthesia in osteoporotic vertebral compression fractures treated with percutaneous kyphoplasty provides better analgesia compared with local anesthesia alone: A randomized controlled study

**DOI:** 10.3389/fsurg.2023.1133637

**Published:** 2023-04-03

**Authors:** Hong-lei Tao, Hang Zhang, Yun-feng Jiang, Shan-shan Fan, Hong-wei Wang, Ao-te Zheng

**Affiliations:** Department of Anesthesiology, Tongde Hospital of Zhejiang Province, Hangzhou, China

**Keywords:** thoracolumbar interfascial block, osteoporotic vertebral compression fractures, percutaneous kyphoplasty, pain management, elderly patients

## Abstract

**Objective:**

To evaluate the safety and efficacy of the thoracolumbar interfascial block (TLIPB) in percutaneous kyphoplasty (PKP), and to confirm that the TLIPB further minimizes perioperative pain and residual back pain on the basis of local anesthesia.

**Method:**

From April 2021 to May 2022, 60 patients with osteoporotic vertebral compression fractures were included in this prospective randomized controlled trial. Patients were randomly assigned to a local anesthesia group (A group) or a TLIPB on the basis of local anesthesia group (A + TLIPB group) before PKP. Pain level (visual analog scale, VAS), amount of analgesic rescue drugs (parecoxib), operative time, mean arterial pressure, heart rate, and complications were assessed and compared between the two groups.

**Results:**

Compared with the A group, VAS scores were lower in the A + TLIPB group, respectively, when the trocar punctured the vertebral body (7.4 ± 0.7 vs. 4.5 ± 0.9; *P* < 0.01), during balloon dilatation (6.6 ± 0.9 vs. 4.6 ± 0.9; *P* < 0.01), during bone cement injection (6.3 ± 0.6 vs. 4.3 ± 0.8; *P* < 0.01), 1 h after surgery (3.5 ± 0.7 vs. 2.9 ± 0.7; *P* < 0.01), and 24 h after surgery (2.5 ± 0.8 vs. 1.9 ± 0.4; *P* < 0.01). Residual back pain (VAS: 1.9 ± 0.9 vs. 0.9 ± 0.8; *P* < 0.01) and the incidence of rescue analgesic use (*P* = 0.02) in the A + TLIPB group were lower compared with the A group. Compared with the A group, mean arterial pressure and heart rate were lower in the A + TLIPB group when the trocar punctured the vertebral body, and with balloon dilatation and bone cement injection; however, there were no statistical differences between the groups 1 and 24 h after surgery. The incidences of bone cement leakage, constipation, and nausea were similar between the two groups. No patient developed infection, neurological injuries, constipation in either group.

**Conclusion:**

The addition of the TLIPB to local anesthesia can further minimize perioperative pain and residual back pain, and reduce perioperative rescue analgesic use. When added to local anesthesia, the TLIPB is an effective and safe anesthetic method for PKP.

**Clinical trial registration:**

This study has been registered in the Clinical Trial registration: ChiCTR-2100044236.

## Introduction

Osteoporotic vertebral compression fractures (OVCFs) are common in elderly patients with osteoporosis (1), which decreases patients' quality of life and results in great economic burden to society (2, 3). With aging of the population, the incidence of OVCFs is increasing annually; 1.4 million patients develop OVCFs worldwide, annually (4). Percutaneous kyphoplasty (PKP) is an effective minimally invasive treatment for patients with OVCFs to stabilize the fracture, effectively restore bone height, relieve pain, and improve patients' mobility (5). However, postoperative residual back pain is a common complication after PKP, with an incidence ranging from 7% to 50% (6, 7).

PKP can be performed under local anesthesia or general anesthesia. Local anesthesia is widely used during PKP (8) because nerve complications caused by vertebral puncture can be easily and quickly identified. However, local anesthetics affect only the skin, subcutaneous tissue, and underlying muscles and provide no anesthetic effect on the vertebral body. Thus, patients may experience intolerable pain and discomfort during balloon inflation and bone cement injection (9). Intolerable pain leads to patients changing their body position during PKP, which may increase the risk of intraoperative spinal cord or nerve injury. Although general anesthesia provides better comfort compared with local anesthesia, elderly patients with OVCFs are likely to have poor underlying health status and poor tolerance for general anesthesia. Furthermore, it is difficult to detect nerve injury during PKP without neurophysiological monitoring. Another problem is that neither local nor general anesthesia can reduce the incidence of postoperative residual back pain. Thus, it is necessary to find an optimal anesthesia method for PKP.

The thoracolumbar interfascial block (TLIPB), a novel regional anesthesia technique, was first described by Hand et al. in 2015 (10). The TLIPB can provide effective analgesia for low back pain by blocking the dorsal rami of the thoracolumbar nerves (11). Previous studies have reported that the TLIPB provided good perioperative analgesia for patients who underwent spinal surgery owing to its simple technique, wide area of analgesia, and minimal contraindications and complications (12). However, to date, few studies have reported the use of the TLIPB in PKP. In this trial, we aimed to evaluate the safety and efficacy of the TLIPB in PKP. We hypothesized that the TLIPB could further minimize perioperative pain and residual back pain, and reduce perioperative rescue analgesic use when added to local anesthesia, and we aimed to confirm that the TLIPB is an effective additional anesthetic method for PKP.

## Materials and methods

### Study design

This prospective, randomized trial was approved by the Medical Ethics Committee of the Tongde Hospital of Zhejiang Province (No. 2020062) before patient enrolment. This trial was registered in the International Clinical Trial Registry (ChiCTR-2100044236). Written informed consent and research authorization were obtained from all subjects participating in the trial.

### Patient cohort

From April 2021 to May 2022, all patients who underwent PKP because of OVCFs were prospectively enrolled in this study. The inclusion criteria were as follows: (1) a single-level OVCF diagnosed by magnetic resonance imaging and bone density testing (*T* score < −2.5); (2) severe back pain associated with an OVCF; (3) compression >15% of the height of the injured vertebra; and (4) injury level from L1 to L5. The exclusion criteria were as follows: (1) compression of the spinal cord and nerve roots; (2) inability to cooperate, such as patients with Alzheimer's disease and other forms of dementia; (3) pathological fractures, such as those associated with vertebral metastatic cancer or osteomyelitis; (4) severe heart disease, or liver or renal failure; and (5) bleeding diathesis associated with the use of anticoagulants or corticosteroids.

Patients were randomly assigned to the local anesthesia group (A group) or A + TLIPB group (in a 1:1 ratio. The random allocation sequence was generated by a computer program and was concealed in opaque, sealed envelopes that were opened 1 day before surgery. The anesthetist was fully aware of each patient's group assignment, while the patients, surgeons, nurses, data controller, and analyst were unaware.

### PKP procedure

All operations were performed by a single anesthetist and a single surgeon in the same laminar air flow operating room. Patients assumed the prone position. Electrocardiographic data, pulse oxygen saturation, respiratory rate, heart rate, and blood pressure were monitored continuously. The affected vertebral pedicles on both sides were identified simultaneously using a C-arm x-ray machine, and the respective positions were marked on the body surface. The lateral projection of approximately 4 mm of the outer edge of the vertebral pedicle was selected as the puncture point.

Patients in the A group received local anesthesia, as follows: 1% lidocaine (40 ml) was injected from the skin to the periosteum of the fractured vertebra. Patients in the A + TLIPB group received the TLIPB, as follows: Under aseptic conditions, a high-frequency linear ultrasound probe (covered by a sterile sheath) was placed vertically at the L3 vertebral level. First, the hyperechoic shadow of the spinous process was visualized as an anatomical guide point. Then, the probe was moved laterally to visualize the paraspinal, multifidus, longissimus, and iliocostalis muscles. The interfascial plane between the longissimus muscle and the multifidus was visualized, and the needle was inserted into the interfascial plane with an in-plane technique in a lateral-to-medial direction. When the needle was inserted into the interfascial plane, 2 ml of normal saline was injected for confirmation. A local anesthetic solution comprising 0.25% bupivacaine was injected bilaterally (20 ml per side, total: 40 ml). Twenty minutes after the TLIPB was performed, sensory testing was performed with the hot-cold test. Local anesthesia was then performed as for patients in the A group.

In all patients, after creating a small skin incision, a bone puncture trocar was gradually inserted at the puncture position and advanced until it reached the lateral margin of the pedicles (10 o'clock on the left side and 2 o'clock on the right side). Next, the puncture trocar was gradually inserted through the pedicles as a working channel until a point 2–3 cm from the trailing edge of the vertebral body was reached, under C-arm guidance. A manual drill was used to enter the vertebral body along the working channel until the end of the bit reached approximately 2–3 mm from the front edge of the vertebral body, and the drill was withdrawn. Then, a balloon was carefully inflated in the vertebral body through the working channel at a depth of 3/4 of the anterior vertebral body. Finally, bone cement was slowly injected into the fractured vertebral body under lateral fluoroscopic guidance.

### Postoperative care

Anteroposterior and lateral plain radiographs were obtained in all patients 1 day after surgery to determine the distribution of the bone cement. Patients used a soft lumbar support belt for 1 month after surgery, resumed routine functional exercise 1 week after the operation, and regularly checked in at the outpatient clinic. All patients took celecoxib 200 g orally twice a day for 1 week. An intramuscular injection of parecoxib (40 mg) was given if a patient reported pain greater than 4 on a 0–10 VAS after celecoxib 200 g was taken, or reported hard to sleep because of pain. A telephone interview was performed 7 days after the operation, and the patients were asked to assess their pain level using a visual analog scale (VAS) score.

### Outcome assessment

The baseline characteristics of the patients comprised age, sex, height, weight, body mass index, and fracture site(s). Pain level and amount of analgesic rescue drugs (parecoxib) were recorded to evaluate the analgesic effect. VAS scores were used to evaluate the level of pain at six time points perioperatively: before surgery (T1), trocar puncture into the vertebral body (T2); balloon dilatation (T3); bone cement injection (T4); 1 h after surgery (T5); and 24 h after surgery (T6). Residual back pain (VAS score) was evaluated 7 days after PKP surgery. We also recorded the operative time, mean arterial pressure (MAP), heart rate (HR), complications of the surgery (infection, bone cement leakage), rescue analgesic use, neurological injuries, and adverse anesthetic events (such as constipation or nausea).

### Statistical analysis

On the basis of the results of previous studies (5), we anticipated an average decrease in VAS (pain) scores of 1.4 in the TLIPB group compared with the A group. With a desired power of 0.90 and significance level of 0.05, a power analysis was performed using PASS 2011 (NCSS, LLC, Kaysville, UT, United States). It was estimated that a sample size of 21 patients per arm was required. With a 20% expected exclusion rate, the minimum sample size was 26 in each group. Therefore, we decided to include 30 patients in each group.

All data analyses were performed using SPSS version 23 (IBM Corp., Armonk, NY, United States). Student's *t*-test or the Wilcoxon–Mann–Whitney *U* test was used to analyze quantitative data, and Pearson's Chi-squared test or Fisher's exact test was used to analyze qualitative comparative data. Statistical significance was defined as *P* < 0.05.

## Results

### Patient demographics

**Figure 1:**
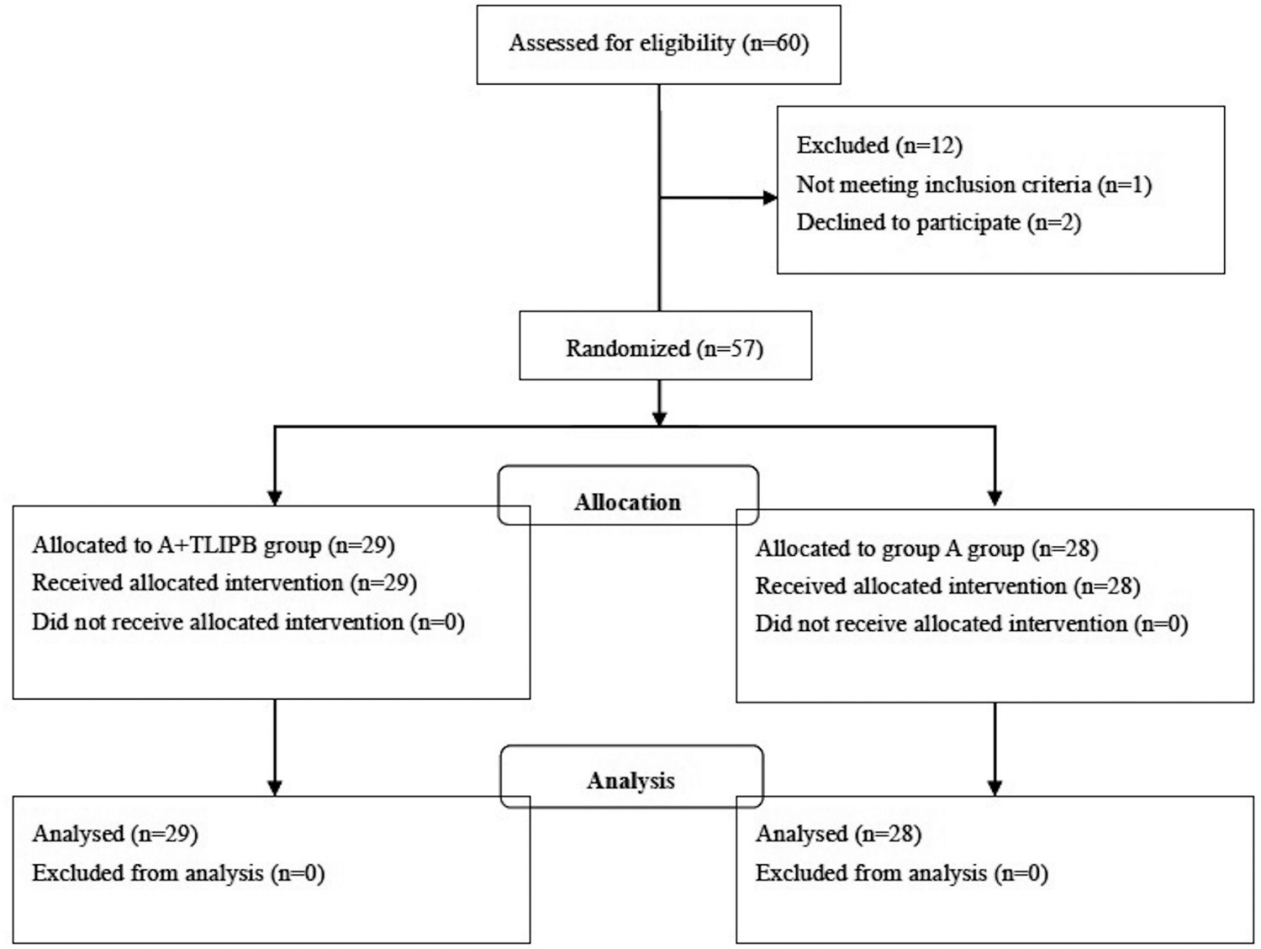
Flow diagram of patients and the study design.

Figure 1 shows the CONSORT diagram of enrollment for this study. From April 2021 to May 2022, 60 patients were scheduled to undergo elective PKP for OVCFs in our institution. Among these patients, one was ineligible and two declined to participate. Thus, 57 patients were included in the final study and analysis; 28 were randomized into the A group and 29 were randomized into the A + TLIPB group (Figure 1). We found no differences in demographic data between the groups (Table 1).

**Table 1:** Baseline characteristics of patients in the two groups.

	A group	A + TLIPB group	*P* value
*n*	28	29	-
Age (years)	78.3 ± 8.7	78.1 ± 9.2	0.94
Weight (kg)	53.7 ± 9.9	57.1 ± 8.1	0.54
Height (cm)	156.9 ± 6.3	157.8 ± 5.2	0.17
BMI	21.8 ± 3.8	22.9 ± 2.9	0.25
Gender (M/F)	4/24	4/25	1
Operative time	33.29 ± 4.90	29.80 ± 3.93	<0.01

### Pain level at the different time points

**Figure 2:**
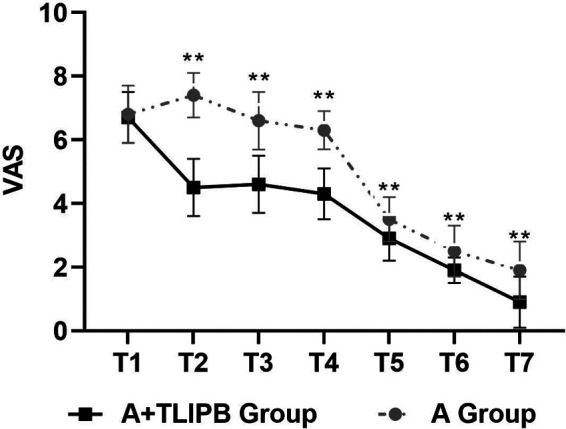
Pain level according to VAS score before surgery, trocar puncture into the vertebral body; balloon dilatation, bone cement injection, 1 h after surgery, 24 h after surgery, 7 days after surgery. The Wilcoxon–Mann–Whitney *U*-test was performed to detect the difference between the groups. **P < 0.001.

There was no statistical difference in preoperative VAS scores between the A group and the A + TLIPB group (6.8 ± 0.9 vs. 6.7 ± 0.8, respectively; *P* = 0.74). Compared with the A group, VAS scores were lower in the A + TLIPB group, respectively, when the trocar punctured the vertebral body (7.4 ± 0.7 vs. 4.5 ± 0.9; *P* < 0.01), during balloon dilatation (6.6 ± 0.9 vs. 4.6 ± 0.9; *P* < 0.01), during bone cement injection (6.3 ± 0.6 vs. 4.3 ± 0.8; *P* < 0.01), 1 h after surgery (3. 5 ± 0.7 vs. 2.9 ± 0.7; *P* < 0.01), and 24 h after surgery (2.5 ± 0.8 vs. 1.9 ± 0.4; *P* < 0.01). Residual back pain in the A + TLIPB group was also lower than that in the A group (1.9 ± 0.9 vs. 0.9 ± 0.8; *P* < 0.01) (Figure 2).

### MAP and HR

**Figure 3:**
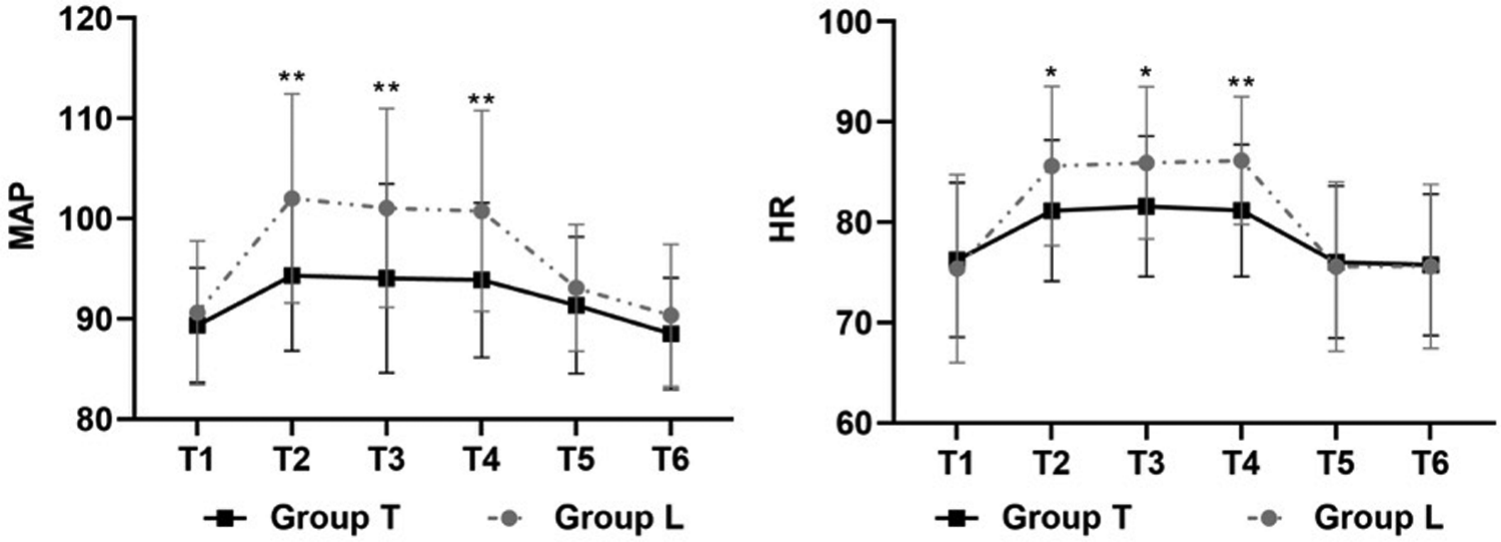
The level of MAP and HR. The Student's *t*-test was performed to detect the difference between the groups. **P < 0.001.

There were no statistical differences in preoperative MAP (90.61 ± 7.19 vs. 89.36 ± 5.72 mmHg; *P* = 0.47) and HR (75.39 ± 9.37 vs. 76.24 ± 7.70 bpm; *P* = 0.71) between the A and A + TLIPB groups, respectively. Compared with the A group, MAP (mmHg) was lower in the A + TLIPB group, respectively, during trocar puncture into the vertebral body (102.01 ± 10.43 vs. 94.32 ± 7.50; *P* < 0.01), during balloon dilatation (101.06 ± 9.91 vs. 94.09 ± 9.44; *P* < 0.01), and during bone cement injection (100.76 ± 10.02 vs. 93.88 ± 7.72; *P* < 0.01); however, there were no statistical differences 1 h after surgery (93.10 ± 6.31 vs. 91.36 ± 6.81; *P* = 0.32) and 24 h after surgery (90.34 ± 7.10 vs. 88.51 ± 5.57; *P* = 0.28). Compared with the A group, HR (bpm) was lower in the A + TLIPB group during trocar puncture into the vertebral body (85.61 ± 7.94 vs. 81.14 ± 7.03; *P* = 0.03), during balloon dilatation (85.93 ± 7.59 vs. 81.59 ± 7.00; *P* = 0.03), and during bone cement injection (86.14 ± 6.37 vs. 81.17 ± 6.58; *P* < 0.01); however, there were no statistical differences 1 h after surgery (75.57 ± 8.43 vs. 76.03 ± 7.58; *P* = 0.83) and 24 h after surgery (75.61 ± 8.17 vs. 75.76 ± 7.06; *P* = 0.94) (Figure 3).

### Complications

Compared with the A group, the incidence of rescue analgesic use was lower in the A + TLIPB group (*P* = 0.02). The incidences of bone cement leakage, constipation, and nausea were similar between the two groups. No patient in either group developed postoperative infection or experienced neurological injuries (Table 2).

**Table 2:** The complications between the two groups.

	A group	A + TLIPB group	*P* value
*n*	28	29	
Infection	0	0	-
Bone cement leakage	3	4	1
Rescue analgesic use	9	2	0.02
Neurologic injuries	0	0	-
Constipation	5	2	0.21
Nausea	2	1	0.53

## Discussion

PKP is an effective minimally invasive treatment for patients with OVCFs to stabilize the fracture, effectively restore bone height, relieve pain, and improve mobility (5, 13). The basic procedure in PKP is the injection of bone cement into the compressed vertebral body to stabilize the fracture and restore vertebral body height. Considering the cost-effectiveness, and the risks of general anesthesia in elderly patients, local anesthesia has been widely used in PKP (14). However, some patients experience severe pain during the procedure under local anesthesia, especially during balloon inflation and bone cement injection, and during advancement of the trocar through the posterior cortical margin (9). Intraoperative pain sensations may trigger cardiovascular events, stress reactions, and negative emotions in patients. Pain sensations may also cause patients to struggle and change their body position (9), which may affect doctors' attention and the operation, and increase the risk of intraoperative spinal cord or nerve injury.

Another associated complication of PKP under local anesthesia is postoperative residual low back pain, which can seriously affect patient satisfaction and reduce the quality of the surgery (15, 16). The incidence of residual back pain after successful PKP surgery was 7.8% in a study by Li et al. (7) and 7.3% in a study by Dohm et al. (6). Previous studies reported that the incidence of lumbar facet joint violation was 9.6% (18), which has been considered as potential source of chronic low back pain (18, 19).

The TLIPB is a type of interfascial plane block, with local anesthetic solution injected into the fascial plane between the multifidus and longissimus muscles at the third lumbar vertebral level to block the dorsal rami of the thoracolumbar nerves. The TLIPB provides an area of analgesia that covers the middle of the vertebra and has a predictable spread from L1 to S1 (20). The dorsal rami run around the facet joints, innervate the surrounding ligaments, joints, and paravertebral muscles, and provide cutaneous sensation from the vertex to the coccyx. The dorsal rami of the spinal nerves innervate the paraspinal muscles and posterior bony elements of the spine, which may explain the analgesic effect of the TLIPB in PKP (21). Blocking the dorsal rami of the thoracolumbar nerves as they pass through the paraspinal musculature could provide effective analgesia for back pain (22). Therefore, the TLIPB may be an effective perioperative pain management technique; thus, we performed this block on the basis of traditional local anesthesia for patients who underwent PKP. In our study, the intra- and postoperative pain scores in the A + TLIPB group were lower compared with the A group, indicating that the TLIPB can further reduce the perioperative analgesia requirement of PKP. Previous studies observed similar results to those in our study, and reported that the TLIPB provided effective analgesia for back pain after spinal surgery (11, 23). The TLIPB can provide longer-lasting analgesia compared with local anesthesia alone, as the absorption of local anesthetic with the TLIPB may be slower because the injection site is an interfascial plane (22). This may be the reason why the TLIPB can provide analgesia to relieve postoperative residual low back pain. As the dorsal rami run around the facet joints, the TLIPB can also relieve chronic low back pain caused by lumbar facet joint violations.

There were several limitations in this study, as follows: (1) the study was a one-side blinded study, because the anesthetist was aware of patients' group assignment. The anesthetist may be biased to preform intraoperative management, which may influence the clinical effect and reduce complication of A + TLIPB group. (2) the follow-up period was only 7 days, the long-term effect of TLIP block was not shown in this study. As residual low back pain may last for a long time, it needs further study to verify whether TLIP block could reduce the residual low back pain after 7 days.

This study showed that the TLIPB offered advantages over traditional local anesthesia alone regarding perioperative analgesia and recovery. Therefore, the TLIPB should be considered a suitable anesthetic technique in patients who undergo PKP.

## Data Availability

The original contributions presented in the study are included in the article/Supplementary Material, further inquiries can be directed to the corresponding authors.
